# Optimal potential of cascading the hydro-power energy generation site, with a hydrokinetic turbine generation system. A case study analysis in the Southern African region

**DOI:** 10.1016/j.heliyon.2024.e39663

**Published:** 2024-10-22

**Authors:** P.B. Ngancha, B.P. Numbi, K. Kusakana

**Affiliations:** a Department of Electrical Engineering, Mangosuthu University of Technology, Durban, South Africa; b Department of Electrical, Electronic and Computer Engineering, Central University of Technology, Bloemfontein, Free State, South Africa

**Keywords:** Hydropower site enhancement, Cascaded configuration, Hydrokinetic setup, Maximum flow rate, Optimal turbine location

## Abstract

The production of hydroelectricity is well-established in many nations worldwide and has a reputation for being resilient, emitting little carbon dioxide, and having a long lifespan. This article begins by examining the hydropower sites capacity in the Southern African region and the potential for generation-site enhancement. The idea is developed from the emergence of new technologies in generating electricity, and the importance of generating more energy in meeting the growing demand. Hence, it is crucial to examine ways to recover the energy that is released shortly after the hydro-turbine rotates when generating energy, hydro flow in the form of kinetic energy of flowing water is released. This article proposes the installation of a hydrokinetic turbine in proximity down flow to the hydro-turbine to recuperate and maximize the surplus energy, using the same water flow. To achieve this goal, a model has been developed with MATLAB-Simulink software, in response to the research gap. This model will calculate the maximum flow rate and, consequently, the optimal location for a turbine in this cascaded configuration. The simulation's results show how adaptable the model is in suggesting a location for the turbine to be placed for maximum power generation. Simulations run in the cascaded hydropower and hydrokinetic setup system, with different scenarios of water volume release from the Dam. As a result, this study provides a sustainable framework for enhancing electricity generation through effective energy recovery while leveraging existing hydropower infrastructure.

## Highlights

• The potential of cascading the hydro-power energy generation sites, with a hydrokinetic turbine generation system.
• A review of the permissible hydro-generation sites in the Southern African region.
• Calculation of the optimal flow rate position in a hydro water flow setup.
• Determination of the optimal placement position of a hydrokinetic turbine, in the cascaded system setup.

## 1 Introduction

In most countries, there is an urgent need for a sustainable and dependable energy supply [1]. The companies that currently produce energy are operating at capacity and are under tremendous pressure to increase demand, and with the demand for clean, sustainable energy, few nations have plans to increase their generating capacities to sustain the rate of growth. Let alone the associated construction delays and costs [2,3]. Countries are searching for alternative energy sources to fill the void as a result, and those in self-sufficient nations are realizing the necessity of storing any excess energy that may have been produced during a period of low demand.

Energy storage is critical because it assists grid operators in meeting their primary goal of assuring continuous and dependable access to power [4]. In Africa, energy utilities and companies, on the other hand, lag in the construction of energy storage systems, as well as in the adoption, learning, implementation, and exploitation of intelligent renewable storage technologies and systems, in responding to electricity requirements [5,6].

There are several types of electrical energy storage methods that are now used in power generating systems across the world; they include battery storage, compressed-air systems, superconducting magnetic energy, pumped-storage hydroelectricity, and so on [7]. Among the many storage methods, a deeper look is aimed at the pumped-storage hydroelectricity method, which is recognized as a method that stores energy in the form of gravitational potential energy of water, pumped from a lower elevation reservoir to a higher elevation [8]. This principle has previously been applied in a variety of ways, including placing an electrical pump, powered during the period when electrical energy is seen to be cheap, and using the cheaper energy to operate the pump, which pumps water from a lower elevation reservoir to a higher elevation reservoir [9]. Although this strategy has shown to be effective over the years, it remains a problem since some energy is required to pump this water to the raised reservoir [9,10].

When considering the capacity issues facing the energy generation today and how the hydroelectric energy generation system functions, it is crucial to recognize the various energy forms (potential and kinetic), found in the hydro-flow cycle. One way to further conserve this energy is to pinpoint the locations where it exists and utilize new technologies to recover and repurpose it. In this research, we will look at utilizing a hydrokinetic turbine, placing it right after the hydroelectric turbine, to pump water from the lower reservoir to the upper reservoir, Hydrokinetic turbines can generate mechanical torque, powerful enough to move a generator shaft while operating at the flow rate of running water. Finding the turbine's optimal scaling flow rate position is necessary for this cascaded installation of the two turbines.

The article main creative idea is a cascade arrangement that aims to increase Southern Africa's hydroelectric power generation by combining hydrokinetic and conventional hydro turbines. This innovative arrangement aims to significantly boost overall energy production by maximizing energy recovery from water flows downstream of hydro-turbines. The MATLAB-Simulink program is utilized to model the ideal turbine location and maximum flow rates within the operational framework, hence emphasizing the novel character of the technology. Finding sustainable sites in the region helps to expand electricity generation and provides a solution to capacity issues. Section II provides a thorough survey of the literature. Section III displays the suggested water flow configuration. Section IV presents the mathematical techniques used to derive the suggested scaling. Section V presents the characteristic of the input data. System modelling in Section VI, the results and discussions presented in Section VII, The results validation are presented in Section VIII, with a conclusion in Section IX.

## 2 Literature review

This review begins by giving a general grasp of the several types of turbines that are frequently used to generate energy. Zhou et al. conducted an analysis on the impact of turbine rotational direction and position on the hydrokinetic turbine array performance. Their findings indicated that the Co-rotating (Co) configuration is the most suitable option for conditions where flow directions are unpredictable. However, counter-rotating-in (CtI) arrangement works better in areas with controlled flow, such as rivers or canals [11]. Khan et al. research work provides a detailed assessment of various turbine systems, including horizontal and vertical axis turbines, and their applications in different water environments, both the horizontal and vertical axis turbines are being explored, with vertical turbines showing more interest in duct augmentation [12]. The impact of an axial-flow hydrokinetic turbine on an erodible channel in both clear water and live-bed situations is examined by Yadav et al. give an In-depth analyses of a range of Hydrokinetic turbines (HKT) design characteristics, to help choose the right HKT for a given set of environmental circumstances, including solidity, power coefficient, angle of attack, number of blades, Tip Speed Ratio (TSR), and performance curves. The article highlights how crucial it is to optimally design these elements in order to improve HKT efficiency and performance under a range of environmental circumstances [13]. The classification of various turbine axis orientations, design types, operating environments, blade designs, and installation techniques is the focus of Septyaningrum et al. research, which led to the identification of crossflow, vertical, and horizontal axis turbines (HAT, VAT) [14]. Most research indicates that crossflow turbines are the most effective for hydrokinetic setup power generation because of their efficiency in shallow water and low flow conditions, their capacity to begin power generation at low flow rates, their ability to adjust their angle to optimize performance in varying flow conditions, and their minimally disruptive design that minimizes the movement of sediment and disturbance of aquatic life.

Combining hydrokinetic energy technologies with cascaded hydropower systems provides a complex but effective way to extract kinetic and potential energy from rivers. To determine and comprehending the maximum flow rate and pinpointing its location along a river system are essential for optimizing energy generation in such systems. Where da Cruz, single out hydrokinetic system and explore flowrate and water velocity in riverine hydrokinetic systems. They concluded that maximizing energy extraction requires positioning turbines in sections of the river were natural flow constrictions or locations with high flow velocities and consistent water movement [15]. Karakaya et al. highlights the challenges of accurately predicting flowrate variations in hydrokinetic systems due to river's conditions and seasonal changes in river discharge. Their study emphasizes the need for advanced modelling techniques and adaptive management strategies to optimize turbine placement [16].

Shibu provide a detailed analysis of flowrate variations in cascading turbine systems, The “Box Jenkins transfer function/noise model” effectively identified the relationship between reservoir inflows and outflows, emphasizing that each subsequent plant in the cascade reduces the flowrate available for downstream turbines due to water withdrawals and energy dissipation through turbines [17]. Li et al. studied flowrate dynamics in multipurpose river systems and found that Hydropower has the highest conversion efficiency among renewable energy sources, with the hydro turbine being the main component. Their findings suggest that optimal flowrate allocation can improve by using turbines that are efficient across a range of flow conditions [18].

In cascaded systems integrated with hydrokinetic generation, Nag and Sarkar developed a framework for determining optimal turbine placement in cascading hydropower and hydrokinetic systems. Their model considers computational fluid dynamics (CFD) models to simulate water flow and optimize turbine placement, identifying zones of maximum kinetic energy potential by running various scenarios to determine the best configuration for maximizing energy output [19]. Wamalwa et al. propose a hybrid approach for assessing flowrate profiles of hydrokinetic energy production and turbine placement. Their study uses a combination of numerical modelling and field data to identify high-velocity locations suitable for hydrokinetic energy extraction [20].

Several methodologies have been proposed for calculating and optimizing flowrates in cascaded hydropower systems combined with hydrokinetic technologies, where Siqueira et al. discuss the application of HEC-RAS for simulating flowrate variations in cascaded systems. Their model identifies potential locations for hydrokinetic turbines based on areas where flow velocity exceeds a minimum threshold for energy extraction. Their procedure involves simultaneous correction of boundary conditions and model parameters, study found an improvement in model performance and reduced error [21]. Hosseini applied AI-based model, compared to computational fluid dynamic (CFD) models to predict maximum flowrate locations in cascaded river systems. Study found that the AI models were over 100 times faster than CFD models and maintained an accuracy within 10 % of the CFD results, and could predict multiple time-steps ahead, reducing dependency on CFD data and enhancing efficiency [22].

Research has further been done on systems involving balancing the needs of upstream and downstream hydropower plants, ensuring that water resources are efficiently used without depleting the flow needed for hydrokinetic turbines. Direct field measurements, such as those made with flow meters or horizontal acoustic Doppler current profilers (H-ADCPs), can also be utilized for calculating flowrate [23]. These instruments enable precise flowrate calculations at certain locations along the river by providing real-time data on water velocity and discharge. In this case, Wang et al. included hydrokinetic turbines into a flowrate optimization model for cascaded hydropower plants. Their methodology considers both hydraulic head and water velocity for turbine placement to maximize total energy output [20].

The literature reveals amongst other things, the lack of standardized criteria for selecting optimal locations for hydrokinetic turbines. The need for advanced modelling techniques to predict flow rate changes and their impact on energy generation output. Research on adaptive turbine designs that can efficiently operate under varying seasonal conditions. Limited research on how hydrokinetic turbines can coexist with other water used turbines, such as hydropower turbine. Calculating maximum flow rates and identifying optimal locations in cascaded hydropower systems with hydrokinetic energy generation involves complex hydrological and engineering considerations. Methods such as the continuity equation, hydrodynamic modelling, and empirical flow data are crucial for accurately predicting flow rates, while factors like river topography, dam placement, and water velocity guide the selection of optimal turbine locations.

Further research is needed to improve the accuracy of flow rate models, especially in the context of the integration of renewable energy technologies. Combining hydropower and hydrokinetic systems presents a powerful strategy for increasing the overall efficiency of water-based energy generation, provided that careful planning and technological safeguards are in place.

To gauge the extent to which the proposed system will contribute to the region's increased generation capacity, relevant studies carried out in the Turkish Antalya Basin, with an emphasis on utilizing a new regional “power duration curve (PDC)" model to estimate the hydropower potential of intermittent rivers. Where the models take advantage of basin features including drainage area, basin relief, and precipitation. To determine which model was best, six were evaluated. High accuracy is demonstrated by the results, with “Nash-Sutcliffe” efficiency values near to 1, signifying accurate power and cease-to-flow point predictions [24]. This research continued to discover and identify the different hydro-generation units that are already in use throughout the Southern African region. The goal is to determine the number of locations and feasibility of implementing these cascaded configurations, as indicated in the table below. Table 1 Lists the hydroelectric stations in Southern Africa, along with information about each one, including its generating capacity, country and location, operation authority responsible for managing day-to-day operations, and a discussion of whether cascaded improvements are possible.

**Table 1:** Hydroelectric power stations in Southern Africa.

Hydroelectric stations in Southern Africa Regone	Countries	Generating capacity (MW and above)	Discussions/Method
Laúca [25]	Angola	2070 MW	The Laúca Hydroelectric Power Plant is the largest operational power plant in Angola. It is situated across the Kwanza River, marking the border between the provinces of Malanje and Cuanza Norte in Angola.
Cambambe [26]	Angola	960 MW	Situated at the boundary of Cuanza Norte and Bengo provinces in Angola, across the Kwanza River, is the hydroelectric power facility known as Cambambe.
Capanda Dam [27]	Angola	520 MW	In the Angolan province of Malanje, the Kwanza River is home to the hydroelectric Capanda Dam. Constructed between 1987 and 2007.
Matala [28]	Angola	40 MW	The Matala power station is located in the Southwest of Angola in the Huíla province, near the town of Matala, along the banks of the Kunene River. Matala is located approximately 175 km, East of the city of Lubango.
Caculo Cabaça [29]	Angola	2172 MW	The power plant is situated in the Cuanza Norte province's village of São Pedro da Quilemba, close to the city of Dondo. It is located in Lunda Norte, Angola, on the Kwanza River/basin. It's roughly 195 km Southeast of Luanda.
Baynes [30]	Angola	600 MW	The hydroelectric power station Baynes is located near Namibia's Northwest border with Angola. Situated at the base of the Baynes Mountains, across the Kunene River, roughly 750 km Northwest of Namibia's capital and largest city.
Dikgatlhong [31]	Botswana	72 MW	The Dikgatlhong dam is situated on the Shashe River, 3 km below the point where it meets the Tati River. The distance between it and the Zimbabwean border is 5 km upstream. Approximately 55 km Northeast of the town of Selebi Phikwe,
Letsibogo [32]	Botswana	120 MW	On the Motloutse River in Botswana, the Letsibogo Dam was constructed with the intention of supplying water for irrigation to the nearby localities and the engineering/manufacturing town of Selebi-Phikwe.
Inga II [33]	Democratic Republic of the Congo (DRC)	1424 MW	The Inga Dams are a pair of hydroelectric Dams that are linked to Inga Falls, one of the biggest waterfalls globally. 140 miles Southwest of Kinshasa, in the Western DRC, Inga Falls are located downstream of Livingstone Falls and the Malebo Pool. At Inga Falls, the average yearly flow rate of the Congo River is roughly 42,000 cubic meters per second.
Inga I [34]	Democratic Republic of the Congo (DRC)	351 MW	Two hydroelectric Dams, called the Inga Dams, are connected to one of the largest waterfalls in the world, Inga Falls. The Inga Falls is situated downstream of Livingstone Falls and the Malebo Pool in the Western DRC.
Nseke [35]	Democratic Republic of the Congo (DRC)	260 MW	Located in the DRC, Nseke Hydroelectric Power Station is a functioning hydropower facility with 260 MW of installed capacity. In Southeast DRC, near the Zambian border, lies the power plant on the Lualaba River in Lualaba province.
Nzilo [36]	Democratic Republic of the Congo (DRC)	108 MW	Nzilo Hydroelectric power station is a functioning hydropower facility. Situated in Lualaba province, in the Southeast of the DRC, near the Zambian border, the power station is situated on the Congo River. The provincial capital city of Kolwezi is located about 30 km to the North of this location.
Zongo 1 [37]	Democratic Republic of the Congo (DRC)	75 MW	In Kasangulu, Bas-Congo Province, there is a power plant called Zongo 1 hydroelectric power plant. Zongo Falls waterfall and Celo-Zongo airport are both close to Zongo 1 hydroelectric power plant.
Mwadingusha [38]	Democratic Republic of the Congo (DRC)	68 MW	The Lufira River in the DRC provides energy for the Mwadingusha hydroelectric power station. In the far Southeast of the DRC, in the town of Mwadingusha, Kambove region, Haut-Katanga Province, the power station is located across the Lufira River.
Piana Mwanga [39]	Democratic Republic of the Congo (DRC)	54 MW	The Piana–Mwanga hydroelectric power station, also known as Mpiana–Mwanga hydroelectric power station. In 1933, the power plant was first put into service to supply a local tin mine. The power plant is situated in the Manono territory of the Tanganyika province of the DRC, across the Luvua River in the town of Piana Mwanga.
Ruzizi II [40]	Democratic Republic of the Congo (DRC)	43.8 MW	Situated on the Ruzizi River, the natural border between the DRC, Rwanda, and Burundi, is the hydropower plant known as Ruzizi II. It is anticipated that the 7.8 MW expansion of the Ruzizi II upgrade will add sustainable power generation.
Ruzizi I [41]	Democratic Republic of the Congo (DRC)	29.8 MW	The Ruzizi River serves as the natural border between the DRC, Rwanda, and Burundi, and is home to the Ruzizi I hydropower plant. and was upgraded to an anticipated increase of 7.6 MW, bringing more generation capacity to the area.
Rutshuru [42]	Democratic Republic of the Congo (DRC)	13.8 MW	The Rutshuru hydroelectric power station, also known as the Rutshuru hydropower station, is situated in the Eastern DRC Rutshuru territory, North Kivu province, and close to the town of Rutshuru. December 16, 2015, marked the completion of the plant.
Barrage de Mobayi-Mbongo [43]	Democratic Republic of the Congo (DRC)	11.37 MW	Originally called Banzyville or Banzystad, Mobayi-Mbongo is a town on the Ubangi River in Nord-Ubangi province, in the Northwest of the DRC. In front of it sits the town of Mobaye in Central Africa. 1989 saw the opening of a tiny hydroelectric power plant.
Centrale hydroélectrique de Sanga [44]	Democratic Republic of the Congo (DRC)	11 MW	Two dams supply the Sanga hydroelectric plant, which is situated in the Kongo Central province, Lukaya District and roughly 20 km upstream of the Zongo Falls.
Kilubi [45]	Democratic Republic of the Congo (DRC)	9.9 MW	The Kiymbi hydroelectric dam and power station was opened in 1959. The Mugandja plateau is the source of the Kyimbi River. The average flow is between 3 and 10 cubic meters per second during the dry season and 150 cubic meters per second during the rainy season.
Centrale hydroélectrique de Kakobola [46]	Democratic Republic of the Congo (DRC)	10.5 MW	Situated in the province of Kwilu in the Southwest DRC, the Kakobola hydroelectric facility is part of the Central Basin. It is situated 790 km East-southeast of Kinshasa and 70 km South-southeast of Kikwit.
Punia [47]	Democratic Republic of the Congo (DRC)	2 MW	The power plant is situated in Manono territory in the small city of Piana Mwanga, across the Luvua River, an outflow of the Congo River.
Centrale hydroélectrique de Lungudi [48]	Democratic Republic of the Congo (DRC)	1.5 MW	Situated in the province of Kasai in the Kinshasa/Gombe DRC, the Lungudi hydropower plant in Tshikapa in the South Western province of kasai. It main water resource is the Lungudi River.
Busanga (China Railway Resources Group) [49]	Democratic Republic of the Congo (DRC)	240 MW	The hydropower project is at Busanga. It is situated in Katanga, DRC, on the Lualaba River/Basin. Located 136 km away from Kolwezi, the mining Center, the Busanga hydropower plant has four turbines.
Kalule [50]	Democratic Republic of the Congo (Cimentkat)	2 MW	The 2 MW Kalule hydroelectric power station located in the upper Congo basin (DRC), is fed by the Kalule Nord river, a right bank affluent of the upper Lualaba.
Lubudi [51]	Democratic Republic of the Congo (Cimentkat)	1 MW	The Lualaba River in the DRC flows into the Lubudi River. Cimenterie du Katanga operates the Lubudi hydroelectric power station, a hydroelectric power plant. Southwest of Kolwezi, the Lubudi rises close to the Zambian border.
Tshiala II [52]	Democratic Republic of the Congo (ENERKA)	6 MW	The ENERKA-operated Tshiala II hydroelectric power plant, also known as the Barrage hydro électrique de Tshiala II, has a 6.00 MW maximum production. Located in East Kasai near Tshiala-Lubilash.
Zongo 2 [53]	Democratic Republic of the Congo (DRC) (SNEL)	150 MW	The DRC Zongo Falls provides energy for the Zongo II hydroelectric power plant. 2018 saw its official launch. In the Central Kongo province in the Southwest DRC, there is a village called Zongo where the Zongo II Dam is situated. The power plant is situated 78 km Southwest of Kinshasa, on the other side of the Inkisi River.
Tshopo [54]	Democratic Republic of the Congo (DRC) (SNEL)	19.65 MW	The 1950s saw the construction of a hydroelectric power plant at the Tshopo river falls, which is in the DRC and to the North of Kisangani. Just before the Lindi River joins the Congo River, it passes through the Northern part of Kisangani. The Tshopo basin covers roughly 17,200 square kilometers.
Kyimbi/Bendera [55]	Democratic Republic of the Congo (SNEL)	17.2 MW	Established in 1959, the Kiymbi Dam is a significant hydroelectric Dam and power plant situated on the Kiymbi River. At a height of 2000 m, the Kyimbi River rises in the Mugandja plateau. It is made up of several falls spaced out over 3.2 km, the biggest of which is 300–500 feet high.
Lutshurukuru [56,57]	Democratic Republic of the Congo (Sominki)	5.6 MW	Located at Pangi, Maniema province, DRC, Central Africa, is the Lutshurukuru hydroelectric power station. The Rutshurur River empties into Lake Mutanda in the Eastern DRC.
Maguga [58]	ESwatini (Eswatini Electricity Company (EEC)	19.8 MW	Approximately 25 m3/sec is released by the Maguga Dam on the Komati River in Swaziland, producing 19 Mw of peak hydropower over two 3-h periods per day on average. A regulating weir elevated to a height of 17 m was necessary to regulate the decreased flow.
Maguduza [59]	ESwatini (Eswatini Electricity Company (EEC)	5.6 MW	The Edwaleni power project is located on the Lusushwana River in Hhohho, Swaziland. The 5,6 MW flow capacity of the hydropower plant is sourced from the Luphohlo Dam reservoir. The Swaziland electricity company manages the plant.
Ezulwini [60]	ESwatini (Eswatini Electricity Company (EEC))	20 MW	The Ezulwini power project is situated on the Lusushwana River, 5.5 km South of Mbabane. The Hydro power plant receives its flow from the Luphohho Dam. In 1988 and 1988, respectively, the first and second units were put into service. The Swaziland electricity company runs its operation.
Edwaleni [61]	ESwatini (Eswatini Electricity Company (EEC))	15 MW	The Edwaleni power project is situated on the Lusushwana River, 5 km West of Manzini Town in Swaziland. The 15 MW design capacity of the hydropower plant is supplied by the Luphohlo Dam Reservoir. The Swaziland electricity company is in charge of its operations.
Muela II [62,63]	Lesotho	32 MW	A transfer tunnel that is 45 km long and connects the Muela reservoir to the Katse Dam. The tail pond that provides hydroelectric power to Lesotho is regarded as the Muela reservoir. A 37-km delivery tunnel leads from the Muela reservoir to the “As River” outfall, where water is sent to the Vaal Dam.
Muela [64]	Lesotho (Butha-Buthe)	72 MW	The “Muela hydropower project” is located roughly halfway between the regional areas of Khukune and Butha Buthe, on the Nqoe River/basin. To balance the flow of the Nqoe River below Muela Tailpond with the flow of the river above the Tailpond, the flow volume is controlled.
Andekaleka [65]	Madagascar (Andekaleka community)	91 MW	Located in eastern Madagascar, close to Andekaleka, on the Vohitra River, is the Andekaleka Dam, a gravity Dam. Water from the Vohitra East is diverted into a 4-km headrace tunnel, which ends at an underground power plant capable of producing 91 MW.
Antelomita [66]	Madagascar (Anjeva Gara)	8.4 MW	Antelomita I and II are the two sections that make up the hydroelectric power plant. On different water falls along the Ikopa River, both are close to one another. It is situated in the rural commune of Anjeva Gara in Madagascar's Analamanga region.
Farahantsana [67]	Madagascar (Mahitsy)	28 MW	Mahitsy commune in the Analamanga is home to the hydroelectric power plant Farahantsana. constructed four penstocks with a diameter of 2500 mm, an intake, a grit chamber, a feeder canal, a headroom chamber, and a run-of-river dam on the Ikopa River.
Mandraka [68]	Madagascar (Mandraka community)	24 MW	The Mandraka Dam, is a gravity Dam located in the Analamanga region of Madagascar on the Mandraka River, close to Mandraka. Constructed In 1956.
Ankevirato [69]	Madagascar (Tsarazaza community)	560 kW	The Ankevirato hydroelectric power station is located near the Tsarazaza, at the Rianamboa River in the Fandriana area, within the Amoron'i Mania region.
Kapichira [70]	Malawi	64 MW	One of the smallest power plants in the Zambezi River basin is the hydroelectric Kapichira Falls hydroelectric facility on the Shire River in Malawi.
Champagne Mauritius [71,72]	Mauritius	30 MW	The Diamamouve Dam, which was built across the Grande riviere, provides water to the Champagne hydroelectric plant. With a 4.3 Mm^3^ storage capacity, the Dam water is transported to the station via an 80m penstock and a 3 km tunnel.
Ferney [73]	Mauritius	10 MW	The Ferney hydroelectric power station, which runs two 5 MW Francis turbines at 600 rpm each, is situated in the eastern region, south of Champagne. They use the waters of “Riviere des Creoles”.
Tamarin Falls [74]	Mauritius	11.383 MW	In the Western region of the nation, Tamarind Falls was founded in 1903. The Dam can hold 2.25 million m3 of water. Water is supplied by two 1.4 km long penstocks. It is located in Mauritius, close to the small fishing community of Henrietta.
Leval [75]	Mauritius	4 MW	Leval hydroelectric is the fourth Dam-based station, having been put into service in 1961. The Eau Bleue reservoir, which has a 4.1Mm3 storage capacity, provides the station with its water.
Cascade Cecile [76]	Mauritius	1 MW	Cascade Cecile, which is situated in the Southern highlands, powers a single Francis turbine that was installed in 1963. Using water from “Riviere des Anguilles”, which is available at 76 m above sea level.
La Ferme [77]	Mauritius	1.2 MW	La Ferme, which was founded in 1959, underwent an upgrade in 1988 when a 1.2 MW Francis turbine with a 1000 rpm gross head was installed. Located on the island of Rodrigues and supply electricity to the locale community.
Cahora Bassa [78,79]	Mozambique	2075 MW	The Cahora Bassa Dam, is one of the Zambezi River's two major Dams, the other being the Kariba. This Dam converts the kinetic energy of the Zambezi River into electricity by turning turbines. The lake's maximum length and width are approximately 250 and 38 km, respectively, and it floods an area of 2700 square kilometers with an average depth of 20.9 m.
Mavuzi [80]	Mozambique (Electricidade de Moçambique)	52 MW	The Mavuzi “Run-of-the-river” hydroelectric power facility, which is situated downstream of the Chicamba hydroelectric power station on the Revue River, close to the Manica province settlements of Maria and Costina. This place is around 132 km southeast of Manica on a road.
Chicamba [81]	Mozambique (Electricidade de Moçambique)	44 MW	The Chicamba hydroelectric power station is a 44-MW hydroelectric power plant that is currently in operation in Mozambique. The power plant is situated in Manica province at Chicamba, across the Ruvue River, close to the international border between Mozambique and Zimbabwe.
Ruacana [82]	Namibia	330 MW	Located across the Kunene River from the town of Ruacana, in Namibia's Omusati region near the Angolan border, is the Ruacana hydroelectric power station. It is by far the largest power plant in Namibia, having been put into service in 1978. The system is operated by “Namibia's national electric power utility company (NamPower)”.
Palmiet [83]	South Africa	400 MW	Two 200-MW (270,000 horsepower) turbine units make up the Palmiet pumped storage scheme, which is situated on the Palmiet River close to Cape Town, 2 km (1.2 miles) upstream of the Kogelberg Dam. During the night, water is pumped to the upper Rockview Dam, using excess power generated by nuclear and conventional coal plants on the grid.
Gariep [84]	South Africa	360 MW	The largest and second-largest water reservoirs in South Africa are the Gariep and Vanderkloof Dams, with Vanderkloof located 130 km downstream of Gariep Dam. They are essential parts of the Orange River water scheme, along with the hydroelectric plants owned by “Eskom”.
Vanderkloof [85]	South Africa	240 MW	The dam was built in 1977 and, when full, has a surface area of 133.43 square kilometers and a capacity of 3187.557 million cubic meters. It is located where South Africa's Northern Cape, Free State, and Eastern Cape provinces meet.
Ingula [86]	South Africa (KwaZulu-Natal)	1332 MW	The Ingula Pumped Storage Scheme, formerly known as Braamhoek, is a pumped-storage power plant situated on the Little Drakensberg range's escarpment, dividing the provinces of KwaZulu-Natal and the Free State. The tunnels link a power plant to the top and lower Dams of the pumped storage hydroelectric project, which are separated by 4.6 km.
Drakensberg [87]	South Africa (KwaZulu-Natal)	1000 MW	The Drakensberg pumped storage scheme is an energy storage facility that allows for the storage of up to 27.6 GW h of electricity in the form of 27 million cubic meters of water. Between Driekloof Dam and Kilburn Dam, electricity generation equipment is located.
Mwenga [88]	Tanzania (Mwenga Hydro Limited)	4 MW	Tanzania's Mwenga Dam is a hydroelectric structure situated in the Iringa Region. Most of the hydropower in the basin is produced by the Kariba and Kafue Gorge dams.
Kidatu [89]	Tanzania (TANESCO)	204 MW	The power plant is situated in the Morogoro Region, in the village of Kilosa, across the great Ruaha River, at a distance of roughly 337 km. Phase II was finished in 1980 and involved the building of two additional 51 MW generators as well as a larger storage Dam, known as Mtera Dam, with a capacity of 3200 million cubic meters.
Kihansi [90]	Tanzania (TANESCO)	180 MW	The Kihansi hydro power station is situated 643 km Southwest of Dar Es Salaam, Tanzania's largest city, across the Kilombero River at the Southern end of the Kihansi Gorge before it converges with the Ulanga River.
Mtera [91]	Tanzania (TANESCO)	80 MW	Tanzania's Mtera Dam is a hydroelectric structure. On the border separating the Iringa and Dodoma regions, the Dam is situated halfway between them. At capacity, it occupies 660 square kilometers. The Great Ruaha River and the Kisigo River feed the 56-km-long and 15-km-wide lake.
New Pangani Falls [92]	Tanzania (TANESCO)	68 MW	Pangani Falls Dam is part of the Pangani hydro systems in Tanzania. The Dam is located at Koani in the Tanga region, Muheza District, about 8 km South of another power station at Hale. The power plant consists of two turbines.
Hale [93]	Tanzania (TANESCO)	21 MW	Located in the town of Hale in the Mnyuzi ward of Korogwe District of Tanga Region, Tanzania, is the hydroelectric Dam known as Hale Dam. It has a 21-MW installed capacity. Tanganiyka had relied on the Pangani River basin as a source of power since the early days of colonization.
Nyumba ya Mungu [94]	Tanzania (TANESCO)	8 MW	The reservoir is located roughly 50 km South of Moshi in the Pangani River valley of the Masai Steppe. The rivers Kikuletwa and Ruvu, which drain an area of about 7500 square kilometers, are its two main sources of water. The largest artificial water body in the Kilimanjaro region, it was constructed in the late 1960s.
Victoria Falls Station B (Underground) [95]	Zambia	60 MW	The Victoria Falls power station, located in Livingstone, Zambia, is a hydroelectric power plant on the Zambezi River. It consists of three power stations and is located in the third gorge below Victoria Falls. Station B, which was built in 1968, has a capacity of 60 MW and six 10 MW machines.
Shiwa Ng'andu [96,97]	Zambia	1 MW	The Shiwang'andu Mini hydro power station is situated 120 km North of Mpika in Siavonga on the Zambezi River. It came about as a consequence of a renewable energy project. of the United Nations Industrial Development.
Old Lunzua [98]	Zambia	0.75 MW	The Lunzua River hydro power station is situated 3 km from the Mbala Mpulungu road and 23 km West of Mbala Town. The power station is located nearly halfway between the towns of Mbala and Mpulungu.
Zengamina [99]	Zambia	0.7 MW	Zengamina is a small hydroelectric power plant located in Northwest Zambia, close to Kalene Hill in the Ikelenge District. Construction took place from 2004 to 2008. Its small hydroelectrical station, which uses the Zambezi River close to its source, provides a reliable electrical supply.
Itezhi-Tezhi [100]	Zambia (Itezhi Tezhi Power Company)	120 MW	The Itezhi-Tezhi Dam on the Kafue River in West-central Zambia was constructed between 1974 and 1977. The Dam floods a portion of Kafue national park, with a height of 62 m and crest length of 1800 m. It creates a reservoir that covers 390 square kilometers.
Mulungushi [101]	Zambia (Lunsemfwa Hydro Power Company)	32 MW	The Mulungushi Dam is built on the Mulungushi River 50 km Southeast of Kabwe, Zambia, with the purpose of supplying hydroelectric power to the Broken Hill Mine and the surrounding community in Kabwe. The Lunsemfwa hydropower company oversees the running of the Dam power plants.
Lunsemfwa [102]	Zambia (Lunsemfwa Hydro Power Company)	15 MW	Situated downstream of the Mulungushi hydroelectric power station in Zambia's Central province, across the Lunsemfwa River, lies the Lunsemfwa Lower hydroelectric power station. Located approximately 143 km north of Lusaka.
Kariba North Bank [103]	Zambia (ZESCO)	1.08 MW	Situated in the Southern province of Zambia, at Kariba, on the banks of the Zambezi River, is the 1.08 GW Kariba North hydroelectric power station. Among them are the 720 MW Kariba North Bank and 360 MW Kariba North bank extension power plants.
Kafue Gorge Upper [104]	Zambia (ZESCO)	990 MW	In connection with the Kafue River in Zambia, the Kafue Gorge upper power station is a 900-MW hydroelectric power plant in operation. 75 km upstream of the Zambezi River confluence with the Kafue River. The river originates in Zambia and travels 2574 km before emptying into eastern Angola.
Kafue Gorge Lower [105]	Zambia (ZESCO)	750 MW	Situated between the Kafue Gorge, upper power station upstream and the Kafue River confluence with the Zambezi River downstream, the power station is situated along the Kafue River. The power plant is situated about 90 km south of Lusaka, the capital of Zambia.
Lusiwasi Lower [106]	Zambia (ZESCO)	12 MW	The Lusiwasi hydropower project has a total capacity of 101 MW. It is built on the Lusiwasi River/basin in central Zambia. Lusiwasi Lake and the Lusiwasi Dam are the main reservoirs.
Victoria Falls Station C [107]	Zambia (ZESCO)	40 MW	located in Livingstone, Zambia, is a hydroelectric power plant on the Zambezi River. Victoria Falls consists of three power stations and is located in the third gorge below Victoria Falls. Station C, which was also built in 1968, has a capacity of 40 MW and four 10-MW machines.
Lunzua [108]	Zambia (ZESCO)	14.8 MW	The Lunzua Hydro power station is situated on the Lunzua River, approximately halfway between Mbala and Mpulungu town, and is 3 km from the Mbala Mpulungu road and 23 km west of Mbala town.
Victoria Falls Station A [107,109],	Zambia (ZESCO)	8 MW	A hydroelectric power facility on the Zambezi River, the Victoria Falls power station is situated in Livingstone, Zambia, and it is made up of three power plants. With two 1 MW machines and two 3 MW machines, Station A, which was constructed in 1936, has an installed capacity of 8 MW.
Chishimba [110]	Zambia (ZESCO)	6.2 MW	The Chishimba Hydroelectric power station is located across the Luombe River from Chishimba Falls. ZESCO advertised the project rehabilitation of the 6 MW power station and expansion to a new 15 MW powerhouse in November 2020.
Musonda Falls I [111,112]	Zambia (ZESCO)	5 MW	The Musonda Falls, hydro power station is 56 km North of Mansa in Luapula province, on the Luongo River. Originally put into service in 1960.
Musonda Falls II [113,114]	Zambia (ZESCO)	5 MW	The Musonda Falls I, hydro power station underwent an upgrade in 2017 from five (5) megawatts to 10 MW, which is now known as Musonda II.
Pungwe A [115]	Zimbabwe	2.1 MW	Pungwe A, hydroelectric power station, situated in Nyamombe River (tributary of the Pungwe River), Honde Valley.
Kariba South [116]	Zimbabwe	1050 MW	The Kariba South power station is located on the South bank of the Kariba Dam that straddles Zambezi River basin between Zambia and Zimbabwe.
Kupinga [113]	Zimbabwe	1.6 MW	The 1.6 MW Kupinga small hydropower station is situated in Chipinge, Manicaland province, Eastern Zimbabwe, on the Rusitu River.
Pungwe B [117]	Zimbabwe	15 MW	Pungwe in the Honde Valley is home to Pungwe B hydroelectric power station. The station is located in the Honde Valley, Manica province, on the Pungwe River.
Pungwe C [118]	Zimbabwe	4.3 MW	Pungwe C hydroelectric power station, obtains its water supply from a weir located in the Honde Valley on the Chiteme River, a Left bank tributary of the Pungwe River.
Tsanga B [114]	Zimbabwe	2.8 MW	Tsanga B hydropower station is supplied by Tsanga River in Nyanga. Situated in Manicaland, Zimbabwe, and provides electricity to Nyanga, a rural community.
Hauna [119]	Zimbabwe	2.3 MW	The Honde Valley's Ngarura River, provides water for the Hauna hydroelectric power station, which generates electricity for the Manicaland province in Eastern Zimbabwe.
Nyamhingura [120]	Zimbabwe	1.1 MW	Nyamhingura hydroelectric power station, is a hydroelectric power station that supplies electricity to the Chimanimani area, Eastern Highlands Estate, Zindi, and Segambe Growth. It receives its water supply from the Nyamhingura River.

**Fig. 1:**
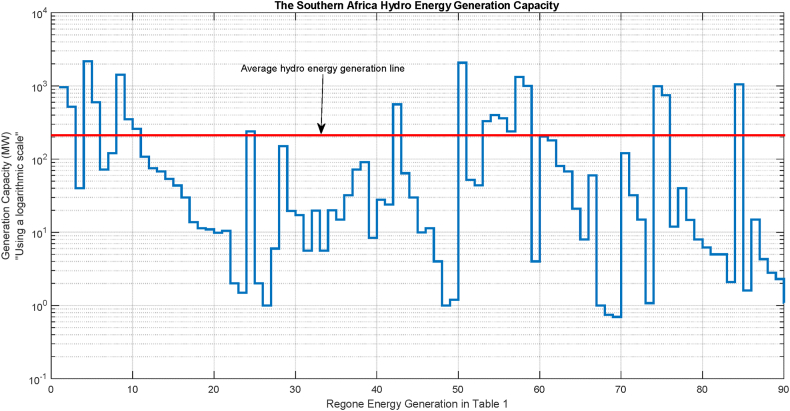
Hydro energy generation sites relative to their capacity, in Southern Africa.

Counting the number of hydroelectric power plants in the 19 countries listed on Table 1, which have a combined generating capacity of almost 22.3 GW and originated from various hydro sources in the Southern African region. The scale of hydro energy generation in each power plant is shown in Fig. 1, with the vertical axis displayed in a logarithmic scale for easier reading. It provides more specific information about an average of approximately 220 MW energy generation capacity of all the generating stations included in Table 1. We can safely say that there is a great deal of room for development in this region. The region is severely lacking in energy, however, by putting these suggested systems into practice and combining a cascading form of hydro-turbine with a hydrokinetic turbine alone a single water flow, ultimately, more energy will be produced.

Several researchers have worked on the sizing parameter of a turbine, where Santolin et al. propose a technique for sizing a small hydropower plant's capacity that takes into consideration both the technical and financial factors. These factors include: Turbine types, the annual energy output, the machine's primary dimensions, the maximum installation height to prevent the onset of cavitation, the installation costs, and two economic factors, namely, the net present value and the internal rate of return. By using the appropriate model equations, the impact of the design conditions on these parameters was assessed. The developed model, when applied to the flow duration curves, enabled identification of the potential design options and comparison of their viability, profitability, and performance [121]. In a separate study, Anagnostopoulos and Papantonis discussed the best kind of turbine for a specific net head and nominal flow rate, recommending a conventional hydraulic turbine with a 50 kW to 10 MW output range, and furthermore, comparing each turbine type's efficiency in relation to the load. According to their findings, a turbine's maximum efficiency varies depending on the kind and size of the device and ranges between 88 % and 91 % for all varieties [122]. Cascaded placement of hydrokinetic and hydropower systems has been implemented in a few instances. Wamalwa et al. work on a system that pumps back some of the downstream water flow and maintains the reservoir's water level, by using a hydrokinetic generating system to power a pump that acts as a pump-back system during the dry season. Their findings indicate a considerable increase in the output of the generator and a 48.18 % reduction in the energy required for pumping [123]. The other cases that may be sited in this paper are cascaded application of hydrokinetic power turbine, in form of turbines array, to improve on the generation capacity [124].

A background on the process of kinetic energy generation in flowing water is provided by the reviews. On a large scale, the review has provided insight into how kinetic energy might be harnessed through the placement of a turbine beneath the stream of water. The outcome in energy generation is mostly dependent on the size and rotation of the blades. On the other hand, the cascading arrangement of a hydro and hydrokinetic turbine feeding from a single water flow channel has not been extensively covered in the literature. Additionally, knowledge of the potential and kinetic energy theories at a falling angle of water, as well as the flow rate, right after the water leaves the hydro-turbine, are still lacking. This study aimed to use a single penstock flow to ascertain the best location for the two turbines, in view of obtaining the optimal output in generation.

## 3 Dam water flow and turbine position

A proposed isolated water catchment system's overall configuration for the cascaded placement position of the hydro and hydrokinetic turbines is shown in Fig. 3. The system consisted of a dam that serves as a catchment area for water, storage reservoir, a powerhouse with two generators powered by hydro-turbine and hydrokinetic-turbine, respectively. The hydro-turbine is positioned in a vertical penstock direction, which is achieved by the damming of the water flow through a sluice gate. Water is received by the hydrokinetic turbine in a horizontal flow movement immediately after the hydro-turbine water is released.

In this configuration, the water from the dam is set up such that it flows vertically into the hydro-turbine and impacts it at an angle. The construction of an embankment, on the other hand, helps with the direction of flow before the hydrokinetic turbine, which helps to maintain or improve the water flow rate. The hydrokinetic-turbine is positioned horizontally to enable the repurposing of the water's kinetic energy flowing from the vertical hydro-turbine.

The average velocity is related to the average transmission of matter (or fluid), it is important to note the restrictions that impact the free flow of water. Hydraulic radius, which symbolizes the ability to physically move flow, is defined as the geographic coordinate linked to the effective depth. The roughness, which is similar to "Manning's Number,” represents all the elements that slow down the flow, including frictional losses, losses brought on by alterations in geometry, and losses related to energy exchange. The longitudinal gradient that indicates the force exerted to move the mass of the plot under consideration is known as the slope of flow [125,126].

## 4 Optimum turbine flow rate mathematical modelling

According to research, we can capture kinetic energy, or moving energy, by harnessing the power of the water flow. Other items, such as a turbine, can be physically moved by this energy flow. The power and energy of flowing water are converted into electricity by turbines in generators [127]. This electricity is then fed into the electrical grid to be used in homes appliances and businesses.

In this work, our attention is focused on two kinds of energy generator, the hydro-power generator turbine and the hydrokinetic power generator turbine. The hydro-turbine converts the energy of falling water into mechanical energy, while the hydrokinetic turbine converts the energy of flowing water into mechanical energy [128,129].

According to the Constaín-Aragón et al., model, the average speed or mean velocity of a falling (or downstream) river will always increase with distance along its channel [125]. Therefore, the energy of falling water may be considered the potential energy, denoted as:

(1) $$P_{e}=V\;.\;\rho_{w}\;.\;g\;.\;h$$

Where, V is the volume of water in litres (L), $\rho_{w}$ is the density of water, g is the acceleration due to gravity and h is the height measured in meters (m). Considering that the hydraulic power at the point of hydro-turbine is proportional to the potential energy, and hydraulic power ($P_{h}$) is denoted as:

(2) $$P_{h}=Q\;.\;P$$

Where, $P_{h}$ is the power in watts, Q is the flow rate in litres per minute (L/min), and P is the pressure in pascal. Obtaining hydraulic pressure, by applying Pascal's Law, which states that pressure, is equal in all points of a confined fluid. Meaning, pressure relates directly to force and inversely to the area in which it excels [130,131]. Knowing that, the force of moving water is the product of the average area, the stream length and distribution coefficient, to the ratio of the time travelled. Therefore, replacing the force of the moving water, equation (2) becomes:

(3) $$P_{h}=\frac{Q\;.\;L\;.\;C}{t}$$

where, L is the stream length in meters (m) and C is the water distribution coefficient and t is the time. Consider the hydro-turbine energy of falling water as the potential energy when water hit the turbine, in a purely vertical position. Therefore, obtaining the hydro-turbine flow rate using equations (1), (3):

(4) $$\frac{Q\;.\;L\;.\;C}{t}=V\;.\;\rho_{w}\;.\;g\;.\;h$$

Equation (4) suggests that the flow rate of the falling water stream is:

(5) $$Q=\frac{V\;.\;\rho_{w}\;.\;g\;.\;h\;.\;t\;}{L\;.\;C}$$

**Fig. 2:**
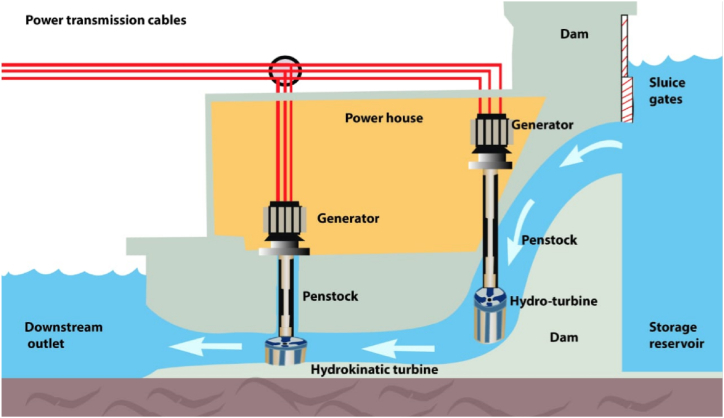
Dam waterfall control channel.

Now that the flow rate of falling water has been determined as shown in equation (5), the focus shifts to calculating the flow rate of a horizontal or running water stream as referred to Fig. 2. Being aware that an object in a horizontal motion can have its kinetic energy computed as:

(6) $$KE=\frac{1}{2}\;.\;m.\;v^{2}$$

where, KE is the kinetic energy measured in joules (J), m is the mass in kilogram (kg) and v is the velocity (or speed) in meter per second (m/s). The kinetic energy of flowing water may be calculated by substituting the mass in equation (6). Therefore, the kinetic energy of flowing water is denoted as

(7) $$KE=\frac{1}{2}\;.\;V.\;v^{2}$$

where, V is the volume of water in litres (L). Considering that the hydraulic power at the point of hydrokinetic-turbine is proportional to the kinetic energy. Hydraulic power is denoted as shown in equation (3), therefore equating the kinetic energy to the hydraulic power, leads to determine the flow rate in a horizontal flow of water:

(8) $$\frac{Q\;.\;L\;.\;C}{t}=\frac{1}{2}\;.\;V.\;v^{2}$$

**Fig. 3:**
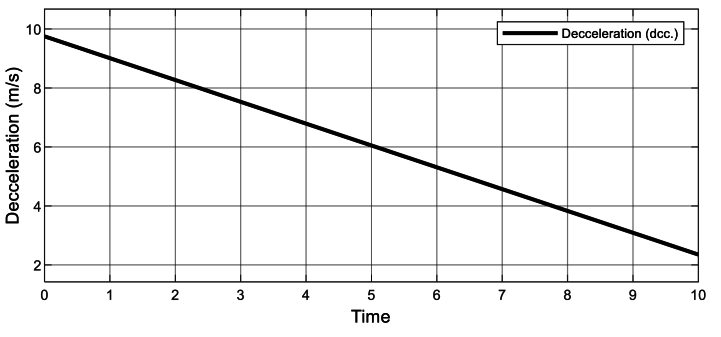
Rate of water flow decelerates.

Since the velocity of flow (v) in normal water downstream cycle is exactly proportional to the flow deceleration and its distance [132]. Replacing the velocity (v) in equation (8), and transposing the equation to determine the flow rate becomes:

(9) $$Q=\frac{V\;.\;D_{cc.\;}\;.\;S\;.\;t\;}{2LC}$$

Where, $D_{cc.\;}$ is the change in water flow deceleration and S is the distance. Considering the significance of comprehending the phenomena linked to water movement in natural flows, the following is an approximation of the average disturbance that affect the velocity of flow in open channels, as suggested by Antoine Chezy and enhanced by Robert Manning for uniform flow [133]:

(10) $$c\approx \frac{R^{\frac{2}{3}}}{n}\;.\;\sqrt{S}$$

Where, c is the water distribution coefficient, n is the Manning Number, R is the hydraulic radius, and S is the flow line's slope in meters (m).

As a result, equation (11) is used to derive the flow rate of running water by substituting equation (10) into equation (9).

(11) $$Q=\frac{V\;.\;\left(\Delta_{dcc.\;}.\;\Delta_{s}\right).\;\Delta_{t}\;}{2LC}$$

At the end, the turbine power $\left(P_{T}\right)$ could be determined as

(12) $$P_{T}=\eta \;.\;\rho_{w}\;.\;g\;.\;h\;.\;Q$$

where, ***η*** is the efficiency of the turbine and ρ is the density of the water.

As a result, it is associated with the energy generated by hydropower or hydrokinetic power, when this mechanical energy from the turbine in equation (12) is applied and connected to the shaft of an electrical generator to produce energy.

## 5 Data characteristics

It is noteworthy that water is one of the high-density matter, categories in the family of renewable energy sources, when compared to gas and air [134]. According to Archimedes principle, the density of water at a particular temperature stays constant throughout the water cycle [135]. It is beneficial in maintaining the turbine's rotation to produce the mechanical energy required to run an electrical generator.

To verify the accuracy of this model, an actual hydroelectric power plant located about 150 km from Bloemfontein is examined and data is gathered from the various equipment datasheets. This is used to perform the simulation and determine the outcome in terms of flow rate capacity and the potential power output, which may be obtained by configuring a hydrokinetic turbine in series with the horizontal water flow of a hydro-turbine. Table 2 shows the parameters of the water channel characteristics selected for this application.

**Table 2:** Specifications of the water flow and turbines.

Water flow channel characteristics	
**Categories**	**Values**
Volume	1000 Ml
River water density	1017kg/ $m^{3}$
Acceleration due gravity	9.8 m/s
Water head height	30 m
Distribution coefficient	0.7
Length	100m
Hydraulic radius [136]	0.17
Manning number [137]	40
Average channel slope [138]	0.01

Developing the previous model to ascertain the optimal location for the flow rate, the quantity of water that is discharged at a time, from the Dam water outlet is referred to as the volume of the water; this amount may stay constant through the hydro turbine and the hydrokinetic turbine. The primary modification in this system is the direction of flow, which is vertical for the hydro turbine and horizontal for the hydrokinetic turbine.

**Fig. 4:**
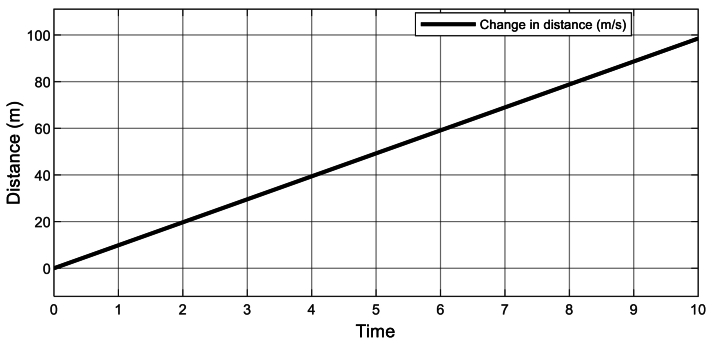
Change in distance of water flow.

Comprehending that, the water flowing to the hydro turbine is impacted by the continuous acceleration that occurs during free fall. The rate, at which the flow decelerates, beginning at its maximum (9.81 m/s) and declining toward the average speed at which running water flow occurs, is further demonstrated in Fig. 3 [139]. This rate is related to the horizontal flow and influences the hydrokinetic turbine flow rate of this simulation. On the other hand, Fig. 4 illustrates the system-estimated change in horizontal flow distance, where the hydrokinetic turbine should be placed.

## 6 Flow rate modeling

**Fig. 5:**
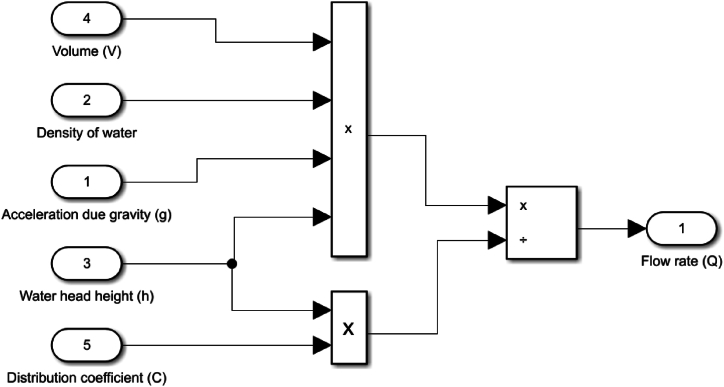
Hydro water flow rate model.

**Fig. 6:**
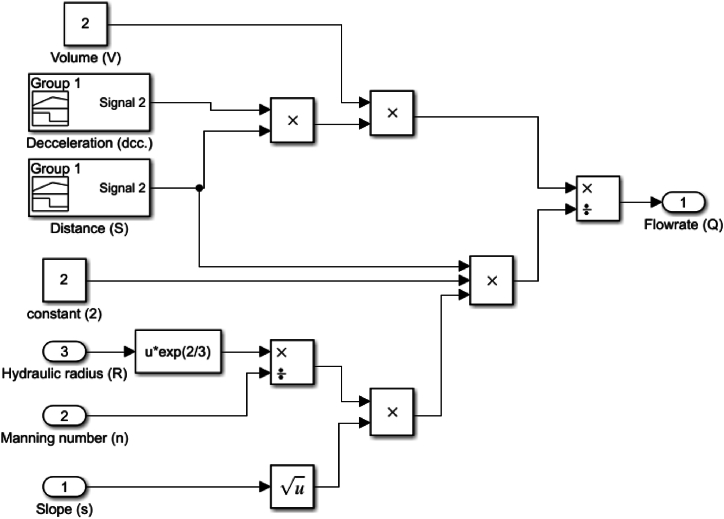
Hydrokinetic water flow rate model.

The hydro-turbine flow rate model is shown in Fig. 5, and the hydrokinetic flow rate model is shown in Fig. 6. The two models are developed using the MATLAB-Simulink tool, which fundamental principle is to offer solvers for modelling and simulating dynamic systems and can export simulated output data [140]. By doing this, the early system design quality can be improved, and fewer faults will be discovered prior to the real physical system being implemented.

## 7 Results and discussions

### 7.1 Turbine optimal position

**Fig. 7:**
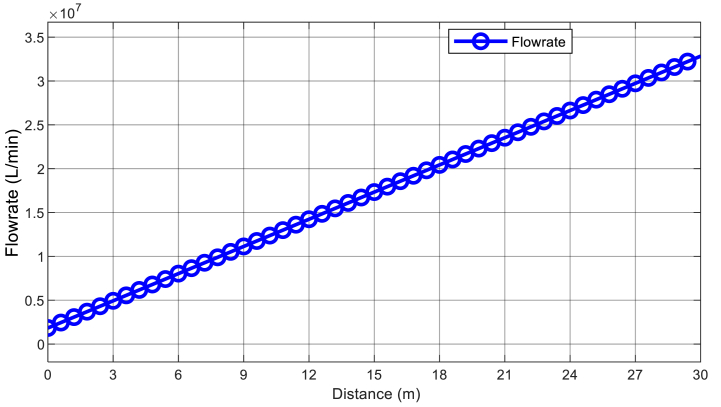
Flow rate of a vertical water channel.

**Fig. 8:**
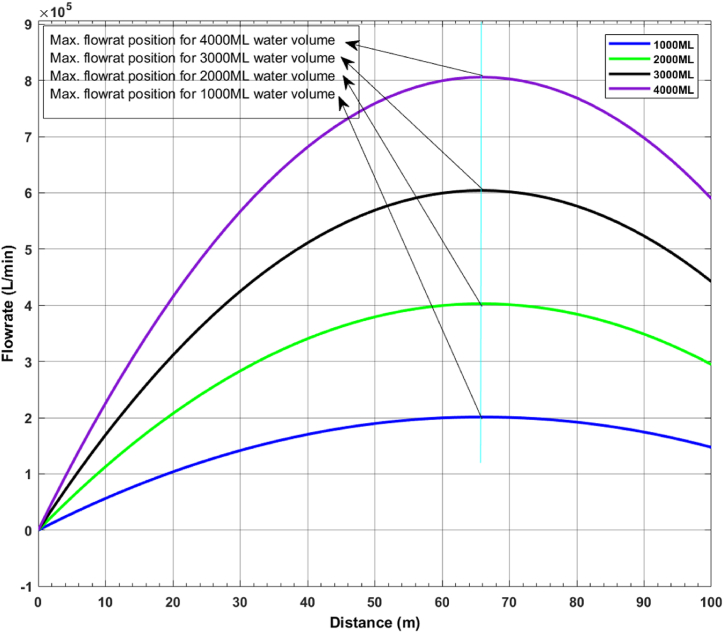
Flow rate of a horizontal water channel.

By utilizing the characteristics listed on Table 2 and using the model developed in Fig. 5, Fig. 6, the results from simulations are obtained, that show, where the turbines should be placed to maximize their efficiency. Fig. 7 shows the potential energy flow rate graph, which serves as a guide for the optimal location of the hydropower turbine. Meanwhile, Fig. 8 shows the flow rate dynamic of the water flow channel, and further indicates where the hydrokinetic turbine should be placed to achieve maximum efficiency.

Fig. 7 output flow rate characteristic demonstrates that, there is approximately zero acceleration due to gravity, when the sluice gate is at it closed position which results in the recording of zero potential energy. It is observed that, the waterfall flow commences precisely when the sluice gate opens, allowing a vertical flow of water to fall toward the hydro turbine. It has been noted that the flow rate rises in direct proportion to the estimated waterfall-to-turbine distance. Thus, the conclusion suggests that the hydro-turbine potential energy increases with distance as long as the flow volume remains constant.

Fig. 8 illustrates the output flow rate characteristics when varying volumes of water (4000 ML, 3000 ML, 2000 ML, and 1000 ML) are utilized. This simulated output validates the model developed for determining the optimal positioning of hydrokinetic turbines for maximum energy generation. As the volume of water released from the dam decreases, the flow rate also changes, demonstrating how different volumes influence the flow characteristics in a horizontal channel. Fig. 8 shows that the flow rate reaches its peak at specific settings: 800 kL/min for 4000 ML, 600 kL/min for 3000 ML, 400 kL/min for 2000 ML, and 200 kL/min for 1000 ML. This demonstrates the correlation between the volume of water and the relevant flow rates that are achieved. The result further reveals that, to optimize energy recovery, the hydrokinetic turbine should be installed at roughly 65.8 m away from the hydro turbine to generate the maximum amount of energy.

## 8 Result validations

The coefficient of variation (cv) formula has been used to validate the simulated results. Several factors, including flow volume and flow distance, have been changed, and the stability of the model has been assessed by comparing the coefficient of variation of various datasets. The relationship between the dataset's mean (μ) and standard deviation (σ) is shown in equation (13) [141].

(13) $$CV=\frac{\sigma }{\mu }X\left(100\right)$$

The standard deviation (σ) of the dataset quantifies the degree of variation or dispersion of a set of values. A low standard deviation suggests that the values tend to be near the mean (average) of the set, whereas a high standard deviation suggests that the values tend to spread out over a wider range. The relationships between standard deviation is shown in equation (14):

(14) $$\sigma =\sqrt{\frac{1}{N}}\sum_{i=1}^{N}{\left(x_{i}-\mu \right)}^{2}$$

Where N is the number of observations in the dataset. $x_{i}$ represents each unique observation, whereas μ is the mean (or average) of the observations.

Table 3 displays the coefficient of variation (cv) result for calculations made using various datasets and flow volumes.

**Table 3:** Stability calculations outcome.

Max. Water released volume	($x_{i}$), Taken from the following distances (m) (20,30,40,50,60,70,80)	Average (μ)	standard deviation (σ)	coefficient of variation (cv)
4000 ML curve	Q(kL/min) = 430,570,682,760,800,805,770	688.14 kL/min	4.1 kl/min	0.59 %
3000 ML curve	Q(kL/min) = 310,423,510,570,600,606,576	513.57 kL/min	3.22 kL/min	0.62 %
2000 ML curve	Q(kL/min) = 208,257,340,380,400,402,384	338.7 kL/min	2.24 kL/min	0.66 %
1000 ML curve	Q(kL/min) = 105,142,170,190,200,200,192	171.28 kL/min	1.04 kL/min	0.61 %

Table 3 presents the validity of the model's results. It includes a calculated average flowrate value for each water released volume sample, as shown in Fig. 8. This average value is used on equation (14) to find the standard deviation of (σ), from which the coefficient of variation (cv) is derived by implementing question (13). The table shows that the estimated coefficient of variation for the flow volumes of 4000 ML, 3000 ML, 2000 ML, and 1000 ML is 0.70 %, 0.22 %, 0.37 %, and 0.77 %, respectively. All readings are less than 1 %, demonstrating the model's adaptability to a variety of applications. However, the chosen datasets show that the model performs significantly better when the flow volume is at 1000 ML, with a relatively low standard deviation and low coefficient of variation.

## 9 Conclusions

This article describes the development of a scalable model that establishes the ideal location for a turbine in a cascaded arrangement of hydropower and hydrokinetic power turbines. An overview of hydropower and hydrokinetic power generation systems was provided at the beginning of the article, and a gap in the utilization of the extra energy released during typical hydropower generation systems was identified. To attend to this challenge, the first step was to determine the optimal position where this energy could be captured, hence, the need to determine the maximum flow rate of each energy type (potential and kinetic) energy in flowing water. After that, a model was developed that considers the various constraints and characteristics. Finally, simulations were run using the MATLAB-Simulink software to ascertain the maximum flow rate, which can be seen in Fig. 7, Fig. 8, for the hydro-power turbine and the hydrokinetic power turbine placement position, respectively, for the best possible energy generation output. The results also show that, in a potential water flow scenario, as the flow distance grows, the maximum flow rate value in a prospective water flow increase. While, in the kinetic water flow position, the hydrokinetic power turbine's maximum flow rate is 800 kL/min for 4000 ML, 600 kL/min for 3000 ML, 400 kL/min for 2000 ML, and 200 kL/min for 1000 ML respectively at 65m, which indicates where the turbine should be positioned to generate the most torque possible for energy generation.

Adding to these outcomes, it should be noted that, although many other elements may potentially affect the flow rate, the main variable that can be used to modify the flow rate in this cascaded configuration is the volume of water released from the Dam through the sluice gate. Therefore, by changing the sluice gate release water volume of the Dam, will change the flow rate, however, the position of maximum flow rate in terms of distance will remain constant, as demonstrated in Fig. 8.

In ongoing research, an effort will be made to determine the hydrokinetic power generation capacity, the amount of energy that could be produced in different cascaded hydropower-hydrokinetic power setups, and their limitations. As a result, an extended perspective will show how much electrical energy the suggested model could be able to produce in the Southern Africa region.

## CRediT authorship contribution statement

**P.B. Ngancha:** Conceptualization. **B.P. Numbi:** Supervision. **K. Kusakana:** Supervision.

## Declaration of competing interest

The authors declare that they have no known competing financial interests or personal relationships that could have appeared to influence the work reported in this paper.
